# Development of an inducible anti-VEGF rAAV gene therapy strategy for the treatment of wet AMD

**DOI:** 10.1038/s41598-018-29726-7

**Published:** 2018-08-06

**Authors:** Christopher A. Reid, Emily R. Nettesheim, Thomas B. Connor, Daniel M. Lipinski

**Affiliations:** 1 Department of Ophthalmology, Medical College of Wisconsin, Milwaukee, WI USA; 2 Nuffield Laboratory of Ophthalmology, University of Oxford, Oxford, UK

## Abstract

Vascular endothelial growth factor (VEGF) is a key mediator in the development and progression of choroidal neovascularization (CNV) in patients with wet age-related macular degeneration (AMD). As a consequence, current treatment strategies typically focus on the administration of anti-VEGF agents, such as Aflibercept (Eylea), that inhibit VEGF function. While this approach is largely successful at counteracting CNV progression, the treatment can require repetitive (i.e. monthly) intravitreal injections of the anti-VEGF agent throughout the patient’s lifetime, imposing a substantial financial and medical burden on the patient. Moreover, repetitive injection of anti-VEGF agents over a period of years may encourage progression of retinal and choroidal atrophy in patients with AMD, leading to a decrease in visual acuity. Herein, we have developed a single-injection recombinant adeno-associated virus (rAAV)-based gene therapy treatment for wet AMD that prevents CNV formation through inducible over-expression of Eylea. First, we demonstrate that by incorporating riboswitch elements into the rAAV expression cassette allows protein expression levels to be modulated *in vivo* through oral supplementation on an activating ligand (e.g. tetracycline). We subsequently utilized this technology to modulate the intraocular concentration of Eylea following rAAV delivery, leading to nearly complete (p = 0.0008) inhibition of clinically significant CNV lesions in an established mouse model of wet AMD. The results shown in this study pave the way for the development of a personalized gene therapy strategy for the treatment of wet AMD that is substantially less invasive and more clinically adaptable than the current treatment paradigm of repetitive bolus injections of anti-VEGF agents.

## Introduction

Age-related macular degeneration (AMD) is the leading cause of blindness in Americans over the age of 65 and effects an estimated 196 million individuals worldwide^1^. AMD is a complex disorder, with multiple factors including age, diet, ethnicity and genetics all contributing to the progression and onset of the disease^2^. Initially, the disease presents in a non-exudative form, typically characterized by pigmentary changes and accumulation of large subretinal deposits, termed drusen, in the macula^3^. In 10–15% of patients, the disease will progress to an exudative form, wherein abnormal vessels from the underlying choriocapillaris invade the subretinal space following the breakdown of Bruch’s membrane, a process known as choroidal neovascularization (CNV). Newly formed blood vessels are frequently leaky, resulting in the buildup of fluid under and within the retina (edema), leading to sudden and irreversible vision loss in approximately 90% of patients. Vascular endothelial growth factor A (VEGF-A) has been identified as a key regulator in CNV progression. As a consequence, the majority of existing therapies function by sequestering and neutralizing VEGF-A through repetitive (i.e. monthly) intravitreal injections of VEGF-A agonists.

Eylea (Aflibercept), a recombinant fusion protein containing the extracellular domains of VEGF receptors 1 and 2 (VEGFR1 and VEGFR2) fused to the fragment crystallization (Fc) region of human IgG1, is commonly used for the treatment of wet AMD due to its strong binding affinity for VEGF_165a_^4^. Current treatment paradigms involve monthly intravitreal injections to reduce exudation and encourage regression of CNV. While effective, repetitive intravitreal injections are associated with numerous complications including endophthalmitis, cataract formation and retinal detachment^5^. Moreover, monthly bolus injections of anti-VEGF drugs may have a significant side effect in which the rate of choroidal, photoreceptor and ganglion cell atrophy is rapidly accelerated^6–8^. It remains unclear whether acceleration of geographic atrophy in AMD patients is the result of an inflammatory response following the trauma of repetitive intraocular injections, or from overdosing of the anti-VEGF agent itself in order to maintain a month-long treatment effect. In either case, in order to improve the outcomes of patients with wet AMD, it is imperative to develop an alternative treatment strategy that reduces the burden placed on the patient while simultaneously minimizing retinal toxicity.

Recombinant adeno-associated viral (rAAV)-mediated gene therapy offers an attractive alternative approach, allowing for long-term transgene expression following a single injection. rAAV-mediated gene therapy has repetitively been demonstrated to be both safe and efficacious at transferring genetic material to the retina in numerous animal models and species, as well as in patients during human clinical trials^9–14^. While great strides have been made in recent years in the design of cell-specific promoters^15,16^ and altering the viral tropism^17,18^, little focus has been given towards the development of technologies to regulate gene expression *in vivo* following transgene delivery.

Currently, the majority of preclinical studies implement gene regulation systems that control expression at the transcriptional level (i.e. inducible promoter systems). These systems typically rely on constitutive expression of a prokaryotic ligand-sensing transactivator protein capable of initiating transcription of a downstream gene of interest. In response to ligand binding, the transactivator changes conformation altering its activity, and in turn altering gene expression^19,20^. While these systems have been shown to be effective at modulating gene expression in small animal models^19,21^, their large size (~1,800 bp) and the unknown immunogenicity of transactivator proteins in humans have limited their use in gene therapy clinical trials.

An alternative approach to regulating gene expression is to do so at the post-transcriptional level by catalyzing degradation of the mRNA transcript in an inducible manner. This can best be achieved through the incorporation of cis-acting RNA elements, termed riboswitches, into the untranslated region (UTR) of the transgene cassette^22,23^. Riboswitches consist of an aptamer domain (ligand sensing element) fused to an expression platform (ribozyme) via a linker sequence and function by altering the structural conformation, and in turn activity of the ribozyme when an activating ligand binds to the aptamer region of the switch.

Importantly, riboswitches are modular as the aptamer domain can be designed to sense small molecules, proteins or ions, allowing for the design of a riboswitch device that responds to potentially any therapeutically relevant activating ligand^24^. Moreover, these switches can also be designed to be either ‘ON-type’ or ‘OFF-type’. In the former, the secondary structure of the ribozyme is in the correct conformation in the absence of the activating ligand, leading to self-cleavage of the mRNA strand and in turn, low basal levels of expression. Ligand binding to the aptamer causes a conformational change in the structure of the ribozyme, causing it to become inactive and preventing self-cleavage, leading to a concomitant increase in mRNA and protein concentrations^25^. The opposite is true for ‘OFF-type’ riboswitches, where the ribozyme domain exists in an inactive conformation in the absence of the activating ligand, resulting in high basal levels of protein expression. Upon ligand binding, the ribozyme will adopt the correct (active) conformation, causing mRNA cleavage and therefore, a decrease in protein production^26^. Favorably, riboswitches do not require any protein co-factors to function and they can easily be incorporated into a rAAV expression cassette due to their small size (~100 bp). While many riboswitches have been designed and tested in mammalian cell culture settings, little is known about how these switches function *in vivo* with respect to baseline expression levels, dynamic range and their ability to induce changes in gene expression following oral dosing of an activating ligand.

Herein, we evaluated the efficacy of five previously published riboswitches, namely K19, L2Bulge9, L2Bulge18tc, GuaM8HDV and TC45, at regulating gene expression in the mouse retina following rAAV-mediated gene delivery^27–31^. After determining the best performing riboswitch in the retina, we demonstrated that rAAV-mediated over-expression of secreted Eylea is able to ameliorate laser-induced CNV formation in a mouse model in an inducible manner, opening the door for a single-injection, life-long, inducible treatment for wet AMD.

## Results

### *In vitro* assessment of riboswitch dynamics

**Figure 1:**
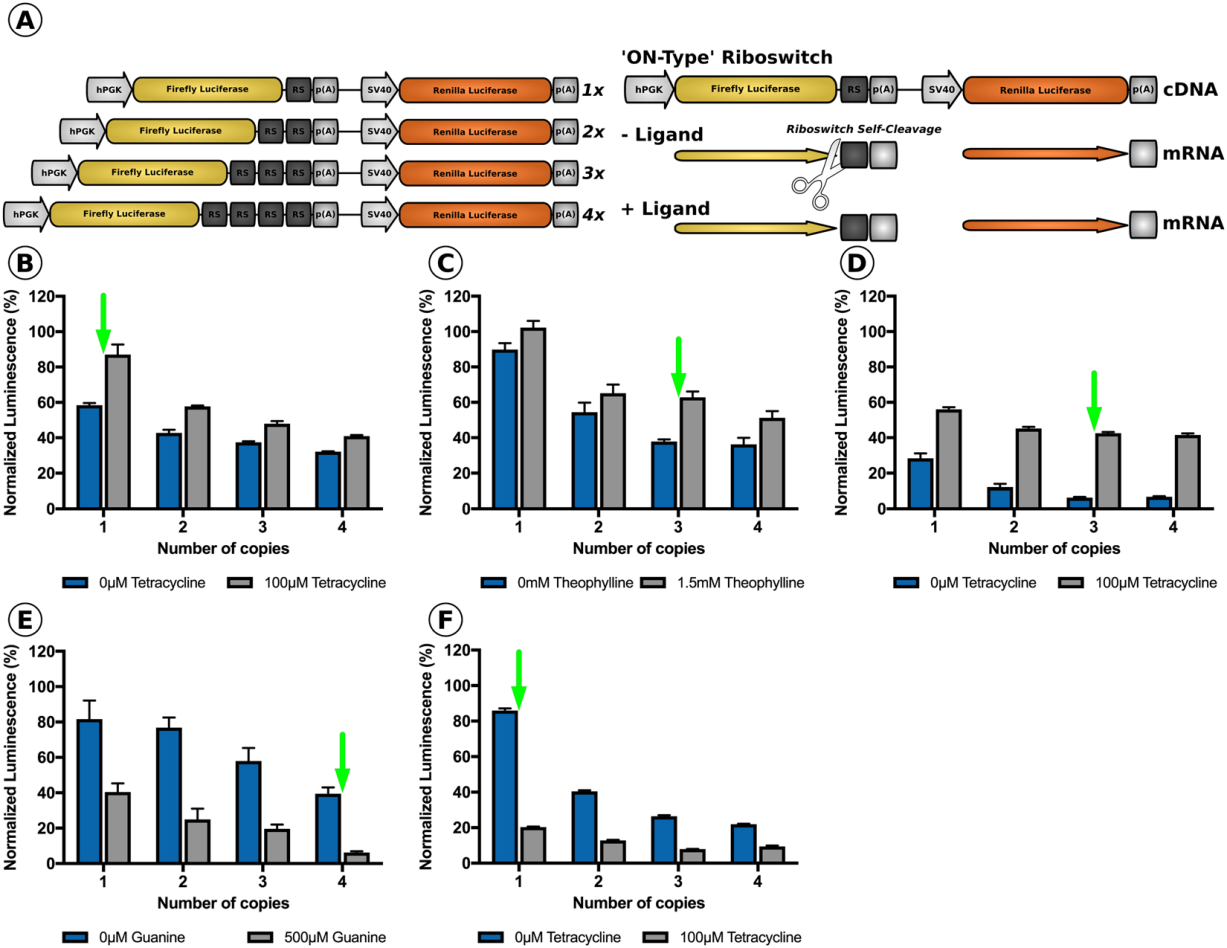
Evaluation of the effect of copy number on riboswitch dynamics. **(A)** Schematic representation of constructs generated through insertion of 1, 2, 3 or 4 copies of each riboswitch into the 3′-UTR and depiction of the mechanism of an ‘ON-type’ riboswitch in the presence or absence of activating ligand. hPGK; human phosphoglycerate kinase promoter, p(A); polyadenylation signal, SV40; Simian vacuolating virus 40 promoter, RS; riboswitch. Dual luciferase assay in HEK293T cells with (grey bars) or without (blue bars) the activating ligand at saturating levels for cells transfected with 1,2,3 or 4 copies of **(B)** K19, **(C)** L2Bulge9, **(D)** L2Bulge18tc, **(E)** GuaM8HDV or **(F)** TC45; *n* = 3 for all groups, green arrow represents the optimal copy number. Values are normalized to unmodified Firefly luciferase activity with standard error shown.

We initially evaluated the ability of three ‘ON-type’ (K19, L2Bulge9 and L2Bulge18tc) and two ‘OFF-type’ (GuaM8HDV and TC45) riboswitches to modulate transgene expression levels *in vitro* following supplementation of their respective activating ligand to the culture media (Supplemental Table 1). Specifically, as inclusion of multiple riboswitch copies within a transgene cassette has the potential to dramatically alter gene expression dynamics^30^, we first sought to determine the number of riboswitch copies required to produce the largest fold-change in transgene expression. To this end, quadruplicate repeats of each riboswitch were synthesized, separated by unique 15 nucleotide barcode spacers that allowed for PCR amplification of a varying number of riboswitch copies (Supplemental Methods **–** A). One, two, three or four copies of each riboswitch were cloned into the 3′-untranslated region (UTR) of a Firefly luciferase reporter transgene; a second luminescent reporter transgene (Renilla luciferase) present in the construct was left unmodified (no riboswitch) served as a transfection control (Fig. 1A). Each of the resulting 20 dual luciferase-riboswitch constructs generated were transiently transfected (*n* = 3 per group) in HEK293T cells either in media lacking the appropriate activating ligand or in the presence of the activating ligand at saturating levels. 24 hours post-transfection, cells were harvested and the levels of Firefly and Renilla luciferase-derived luminescence were recorded using a plate reader. Increasing the riboswitch copy number for both ‘ON-type’ and ‘OFF-type’ riboswitches resulted in decreased basal activity (Fig. 1B–F). K19, a tetracycline responsive ‘ON-type’ riboswitch produced the largest fold-change in luminescence when one copy of the riboswitch was included in the dual-luciferase reporter construct (Fig. 1B), while the other ‘ON-type’ riboswitches, L2Bulge9 (theophylline responsive; Fig. 1C) and L2Bulge18tc (tetracycline responsive; Fig. 1D) achieved the largest fold-change when three copies were included in the cassette. The two ‘OFF-type’ riboswitches tested also had varying optimal copy numbers. GuaM8HDV, a guanine responsive Hepatitis Delta derived switch, generated the largest fold-change in luminescence when four copies were included in the 3’-UTR of the Firefly luciferase transgene (Fig. 1E), while TC45, a tetracycline responsive switch required only one copy of the riboswitch to produce the largest dynamic range (Fig. 1F).

**Figure 2:**
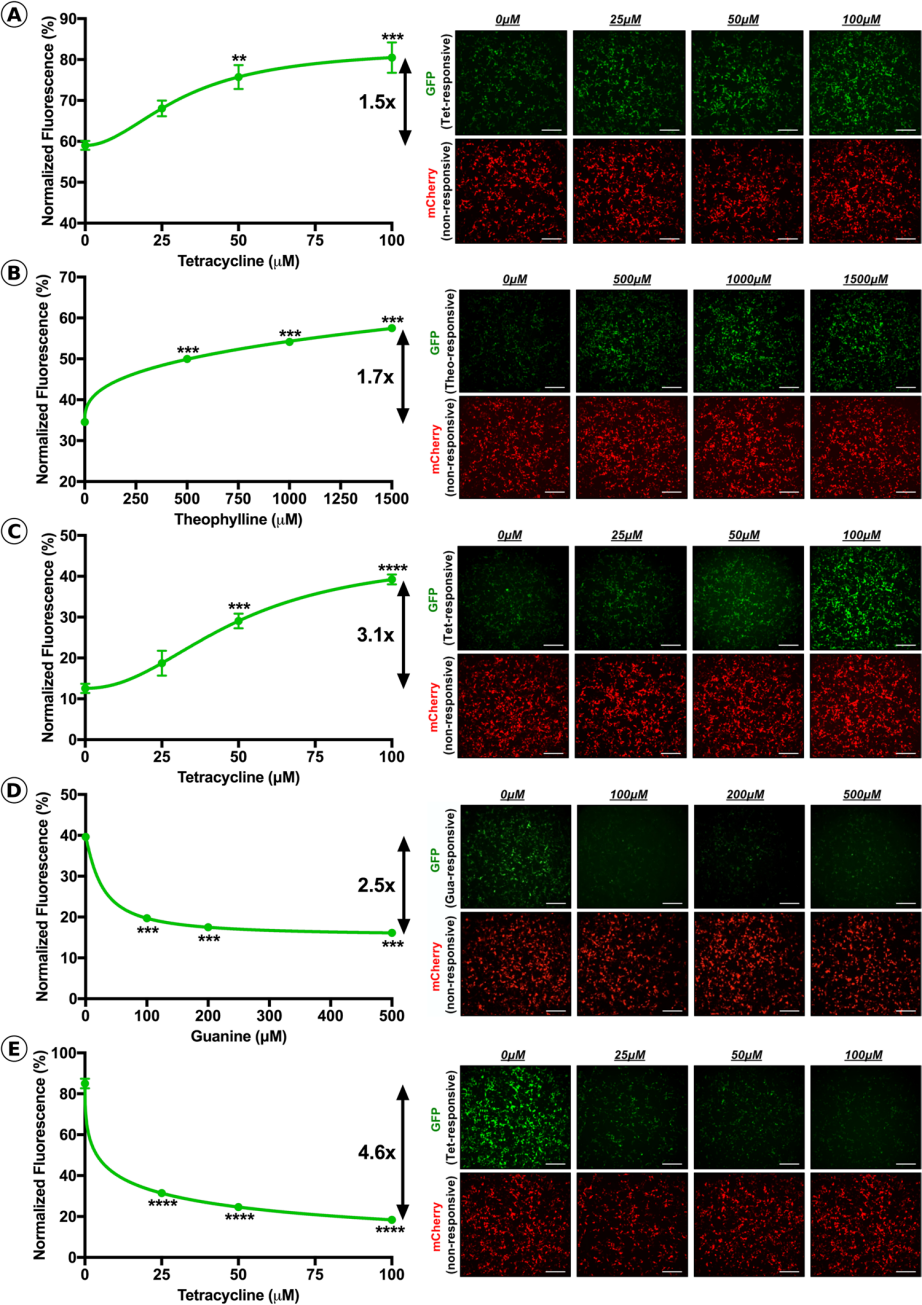
Assessment of riboswitch dosing kinetics in HEK293T cells. Normalized GFP fluorescence and representative GFP (inducible) and mCherry (non-inducible) images at varying concentrations of activating ligand for **(A)** K19, **(B)** L2Bulge9, **(C)** L2Bulge18tc, **(D)** GuaM8HDV and **(E)** TC45. Dynamic range is represented as fold-change. *n* = *3*, **p > 0.01, ***p > 0.001, ****p > 0.001, One-Way ANOVA with Tukey’s post-hoc test. For each group, respective fluorescent channels were recorded with constant exposure and gain; scale bars = 50 μM.

In order to determine the dose-responsiveness of the selected riboswitches, the optimal copy number of each riboswitch were individually cloned into the 3′-UTR of a ubiquitously expressing rAAV GFP reporter cassette, allowing cellular fluorescence to be used as a measure of riboswitch activity. Each of the riboswitch-inducible GFP reporter constructs were co-transfected into HEK293T cells with a non-inducible ubiquitously expressing mCherry construct in the absence or presence of the activating ligand over a range of concentrations (*n* = *3*, all groups). Fluorescent images were obtained for both GFP and mCherry prior to quantification of relative fluorescence using a plate reader. Inclusion of one-copy of the tetracycline responsive K19 switch in the GFP expression construct resulted in a small, but statistically significant dose-dependent increase in GFP expression levels at concentrations of tetracycline 50 μM or higher (*p* < *0*.*05*, One-Way ANOVA), with an overall dynamic range of 1.5-fold (Fig. 2A). Inclusion of three copies of the theophylline responsive riboswitch, L2Bulge9, into the GFP expression cassette led to a significant increase in GFP fluorescence at theophylline concentrations of 500μM and above (*p* < *0*.*001*, One-Way ANOVA), resulting in a maximum dynamic range of 1.7-fold (Fig. 2B). The L2Bulge18tc riboswitch generated the largest dynamic range (3.1-fold, Fig. 2C) observed for an ‘ON-type’ switch, resulting in a significant increase in normalized GFP expression at concentrations above 50 μM (*p* < *0*.*001*, One-Way ANOVA). As expected, the inclusion of either ‘OFF-type’ riboswitches, 4×-GuaM8HDV or 1×-TC45, led to significantly decreased GFP expression from basal levels in the presence of > 100 μM guanine (*p* < *0*.*001*, One-Way ANOVA) or >25 μM tetracycline (*p* < *0*.*0001*, One-Way ANOVA), respectively (Fig. 2D,E). The overall dynamic range of the 4×-GuaM8HDV construct was 2.5-fold, while 1×-TC45 generated the largest dynamic range of all riboswitches tested at 4.6-fold compared to baseline.

### *In vivo* assessment of riboswitch dynamics in the mouse retina

**Figure 3:**
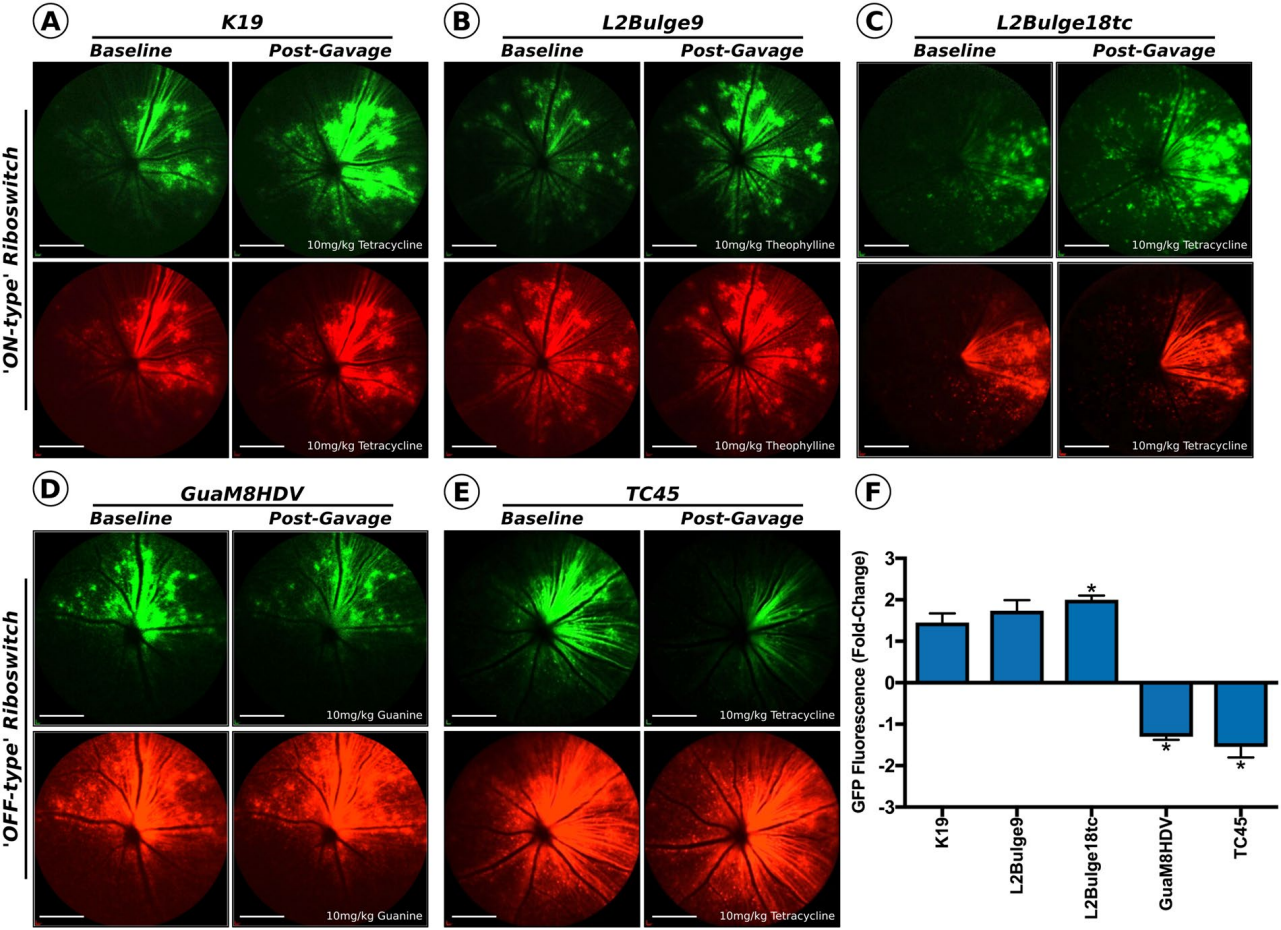
Riboswitch mediated gene modulation in the mouse retina following rAAV gene delivery. Four weeks following intravitreal delivery, baseline GFP and mCherry images were recorded. Seven days following baseline imaging mice received a 10 mg/kg gavage of the activating ligand and were re-imaged 2 hours post-gavage. Representative GFP (inducible) and mCherry (non-inducible) images for **(A)** K19, **(B)** L2Bulge9, **(C)** L2Bulge18tc, **(D)** GuaM8HDV and **(E)** TC45 are shown; Scale bars = 200 nm. **(F)** Quantified post-treatment GFP fluorescence normalized to mCherry fluorescence and displayed as a change from baseline levels (set at 100%). *n* = 3–7, *p > 0.05, paired *t* test.

Although the activity of these riboswitches has been studied extensively in a cell culture setting, many have yet to be evaluated in an animal model. Indeed, accurately quantifying riboswitch dynamics *in vivo* presents many challenges, namely delivery of the construct to the target cell type and accurate quantification of changes in transgene expression *in vivo*. The retina provides a unique environment in which to quantify changes in transgene expression, allowing for the direct visualization of the retina and the repetitive measurement of relative levels of cellular fluorescence using non-invasive confocal scanning laser ophthalmoscope (cSLO) imaging. To this end, inducible GFP reporter constructs under the control of each riboswitch (1×-K19, 3×-L2Bulge9, 3×-L2Bulge18tc, 4×-GuaM8HDV or 1×-TC45) were packaged in rAAV2/2 and co-injected intravitreally (1.2 × 10^9^ vg/eye) with a rAAV2/2 vector packaging a non-inducible mCherry reporter construct (1.2 × 10^9^ vg/eye) (*n* = 5 eyes per group, Supplemental Fig. 1). Four weeks post-injection, a custom-built dual-color multiline cSLO (see methods for instrument details) that allows for concurrent and independent visualization of red or green wavelength fluorophores was used to obtain baseline measurements of mCherry (561 nm) and GFP (486 nm) expression for each group (Fig. 3A–E). Seven days post-baseline imaging, animals received a high dose of the activating ligand (10 mg/kg) via oral gavage. Two hours post-gavage (determined to be the time point in which the highest level GFP expression occurs for ‘ON-type’ riboswitches, with expression returning to baseline levels ~10 hours post-gavage – data not shown) mCherry and GFP fluorescence fundus images were rerecorded for each animal (Fig. 3A–E). By normalizing the fluorescence intensity of the inducible-GFP images to the fluorescence intensity of concurrently captured non-inducible mCherry images (see methods for details on image normalization and quantification) we were able to accurately quantify the relative changes in GFP protein expression in the retina in response to ligand dosing and subsequent riboswitch activation (Fig. 3F). While the dynamic range observed *in vivo* for each ‘ON-type’ riboswitch closely correlated to the levels observed in cell culture, only the 3×-L2Bulge18tc riboswitch demonstrated a significant increase (2-fold) in GFP expression compared to baseline levels *in vivo* (*p* < *0*.*05*, paired *t* test). The activation of the two ‘OFF-type’ riboswitches (4×-GuaM8HDV and 1×-TC45) led to significantly decreased GFP expression from baseline levels following oral gavage of the ligand (*p* < *0*.*05*, paired *t* test), but the dynamic range did not reach levels seen in cell culture, potentially due to minimal turnover of the GFP (~26 hr half-life) during the two-hour period between ligand administration and assessment.

**Figure 4:**
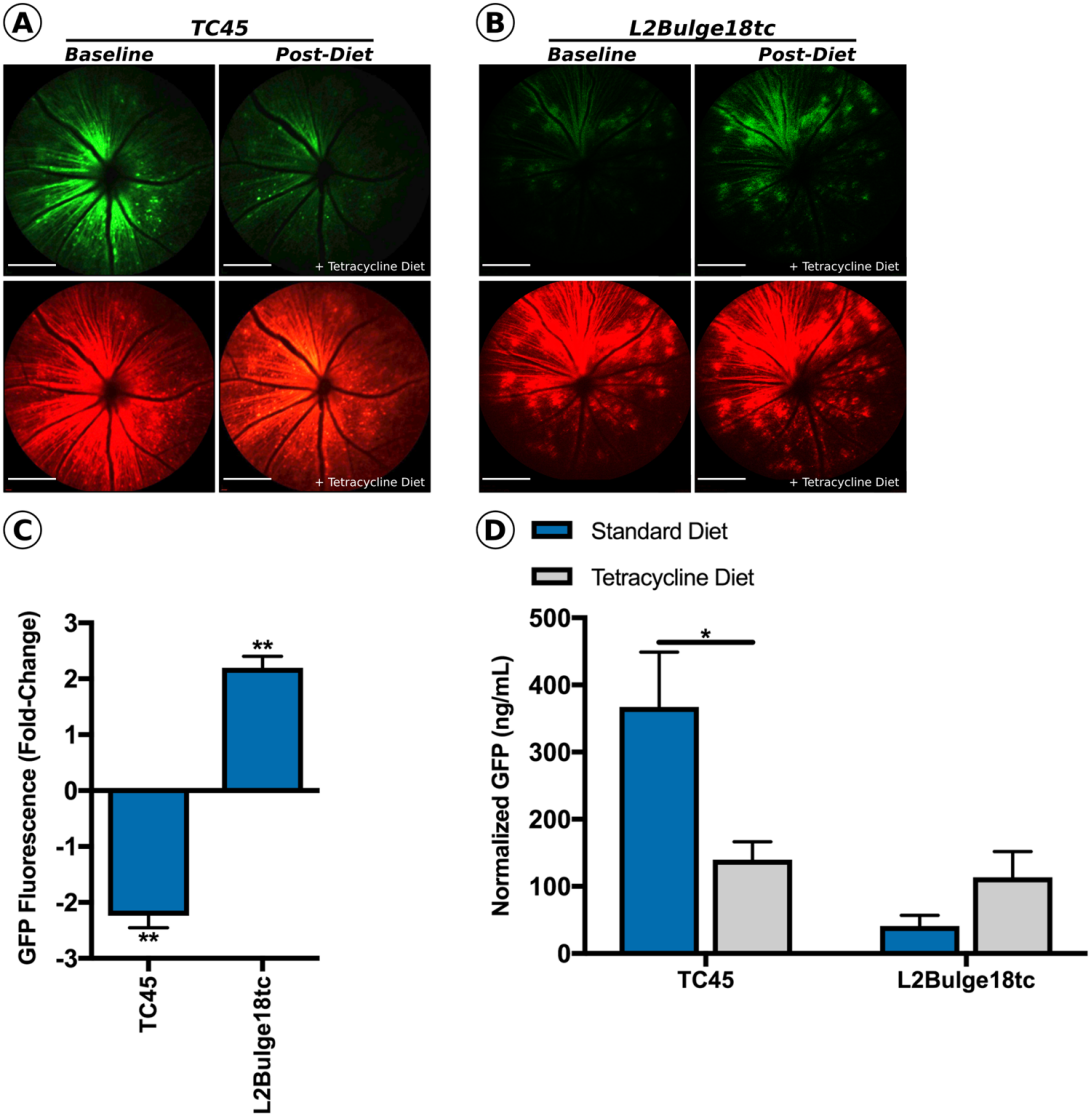
Persistent dosing improves dynamic range in smCBA.GFP-1×-TC45 and smCBA.GFP-3×-L2Bulge18tc treated mice. Following baseline imaging, mice were provided with tetracycline diet *ad libitum* for 7 days at a concentration of 50 g/kg. Representative GFP and mCherry baseline and post-treatment fluorescent images of mice injected with **(A)** smCBA.GFP-1×-TC45 or **(B)** smCBA.GFP-3×-L2Bulge18tc. **(C)** Quantified post-treatment GFP fluorescence normalized to mCherry fluorescence and represented as percentage of baseline GFP fluorescence. *n* = 6, **p > 0.01, paired *t* test. **(D)** Normalized GFP protein levels in smCBA.GFP-1×-TC45 or smCBA.GFP-3×-L2Bulge18tc injected mice receiving tetracycline or standard diet for 4 weeks. *n* = 3, *p > 0.05, unpaired *t* test.

In order to determine if the dynamic range observed *in vivo* could be improved upon by providing the activating ligand over an extended time period, 2.4 × 10^9^ vg of vector packaging the best performing ‘ON-type’ (rAAV2/2.smCBA-GFP-3×-L2Bulge18tc) or ‘OFF-type’ (rAAV2/2.smCBA-GFP-1×-TC45) riboswitch were co-injected intravitreally with 2.4 × 10^9^ vg of non-inducible mCherry reporter vector (rAAV2/2.smCBA-mCherry; *n* = 7 eyes per group). Four weeks following injection, baseline mCherry and GFP fluorescent images were recorded using cSLO imaging (Fig. 4A,B) and mice were subsequently placed on diet containing 50 g/kg of tetracycline, the activating ligand for both switches. Following one week of constant tetracycline dosing through dietary supplementation, GFP and mCherry images were re-recorded (Fig. 4A,B) and GFP fluorescence was quantified as previously described. As expected, prolonged tetracycline mediated activation of the TC45 riboswitch resulted in a significant decrease in GFP fluorescence (2.2-fold) from baseline levels (*p* < *0*.*01*, paired *t* test; Fig. 4C). Eyes injected with vector containing the ‘ON-type’ L2Bulge18tc riboswitch showed significantly increased GFP fluorescence (2.2-fold) from baseline levels (*p* < *0*.*01*, paired *t* test; Fig. 4D). Interestingly, continuous dosing (seven days) resulted in a negligible increase in dynamic range for the ON-type switch when compared to single bolus dosing, likely reflecting the ability of transduced cells to manufacture GFP protein rapidly following transgene activation.

### *In vivo* quantification of changes in fluorescence by dual-color cSLO imaging correlates strongly with changes in protein concentration

Although *in vivo* cSLO imaging has been used to quantify changes in fundus autofluorescence in both humans and animal models^32,33^, its use to accurately quantify changes in ganglion and inner retinal cell derived GFP fluorescence has yet to be validated. Furthermore, as this method prohibits the measurement of the absolute dynamic range (i.e. change in protein levels caused by riboswitch activation), we sought to determine if changes in total protein concentration correlated with changes in fluorescence, as detected through *in vivo* cSLO imaging, in order to determine the absolute dynamic range of the best performing ‘ON and OFF-type’ switches tested.

To this end, wildtype C57BL/6 J mice *(n* = 6 eyes) were co-injected with either rAAV2/2.smCBA-GFP-1×-TC45 or rAAV2/2.smCBA-GFP-3×-L2Bulge18tc and non-inducible AAV2/2.smCBA-mCherry (2.4 × 10^9^ vg per vector; 4.8 × 10^9^ vg per eye), in line with previous experiments. Immediately following injection, mice (n = 3 per group) were randomly assigned to receive either custom tetracycline-containing diet or standard diet. Four weeks post-injection, eyes were harvested and GFP and mCherry protein concentration was quantified using GFP- and mCherry-specific enzyme linked immunosorbent assays (ELISA; Abcam, Cambridge, UK). ‘OFF-type’ rAAV2/2.smCBA-GFP-1×-TC45 injected eyes from mice placed on tetracycline diet had significantly decreased GFP protein concentration (−227.4 ng/mL; 2.6-fold decrease) compared to mice consuming standard diet (367.2 ng/mL, *p* < *0*.*05*, unpaired *t* test; Fig. 4D). As expected, being placed on tetracycline diet resulted in an increase in GFP protein concentration in eyes injected with ‘ON-type’ rAAV2/2.smCBA-GFP-3×-L2Bulge18tc (+72.5 ng/mL; 2.7-fold increase) compared to mice receiving standard diet (41.1 ng/mL, *p* > *0*.*05*, unpaired *t* test; Fig. 4D). Importantly, changes in GFP protein levels for both riboswitches tested were normalized to the concentration of non-inducible mCherry protein and were found to correlate strongly with changes in GFP fluorescence measured using dual-color cSLO imaging. As a consequence of this preliminary screening, the TC45 riboswitch was deemed to be the best performing switch by virtue of having the greatest dynamic range and was selected for use to control expression of Eylea in all subsequent experiments.

### Riboswitch-mediated intraocular tuning of Eylea alters the severity of CNV formation

A rAAV transgene cassette was designed to include a ubiquitous promoter (smCBA), a codon-optimized Eylea cDNA sequence containing a secretion signal adapted from Interleukin-6 and a strong polyadenylation signal (Supplemental Fig. 2A). As murine Vegf-A is only 87% homologous to human VEGF-A, we initially sought to determine if Eylea is capable of binding and sequestering the murine isoform of the protein. In order to validate that our construct produced high levels of secreted Eylea capable of inhibiting murine Vegf-A, we employed a Vegf-specific ELISA, wherein Vegf bound to Eylea cannot be detected (Supplemental Fig. 2B). Recombinant murine Vegf_164_ was spiked in media recovered from HEK293T cells transfected with either an Eylea expression construct or an empty plasmid (*n* = *3*). Following a brief incubation period (30-minutes), unbound Vegf_164_ protein levels were assessed (Supplemental Fig. 2C). Eylea expression resulted in a significant decrease in detectable Vegf_164_ protein (i.e. unbound protein) compared to mock transfected cells (*p* = 0.0007 unpaired *t* test), confirming the construct produces secreted, biologically active Eylea capable of sequestering the mouse ortholog of VEGF.

**Figure 5:**
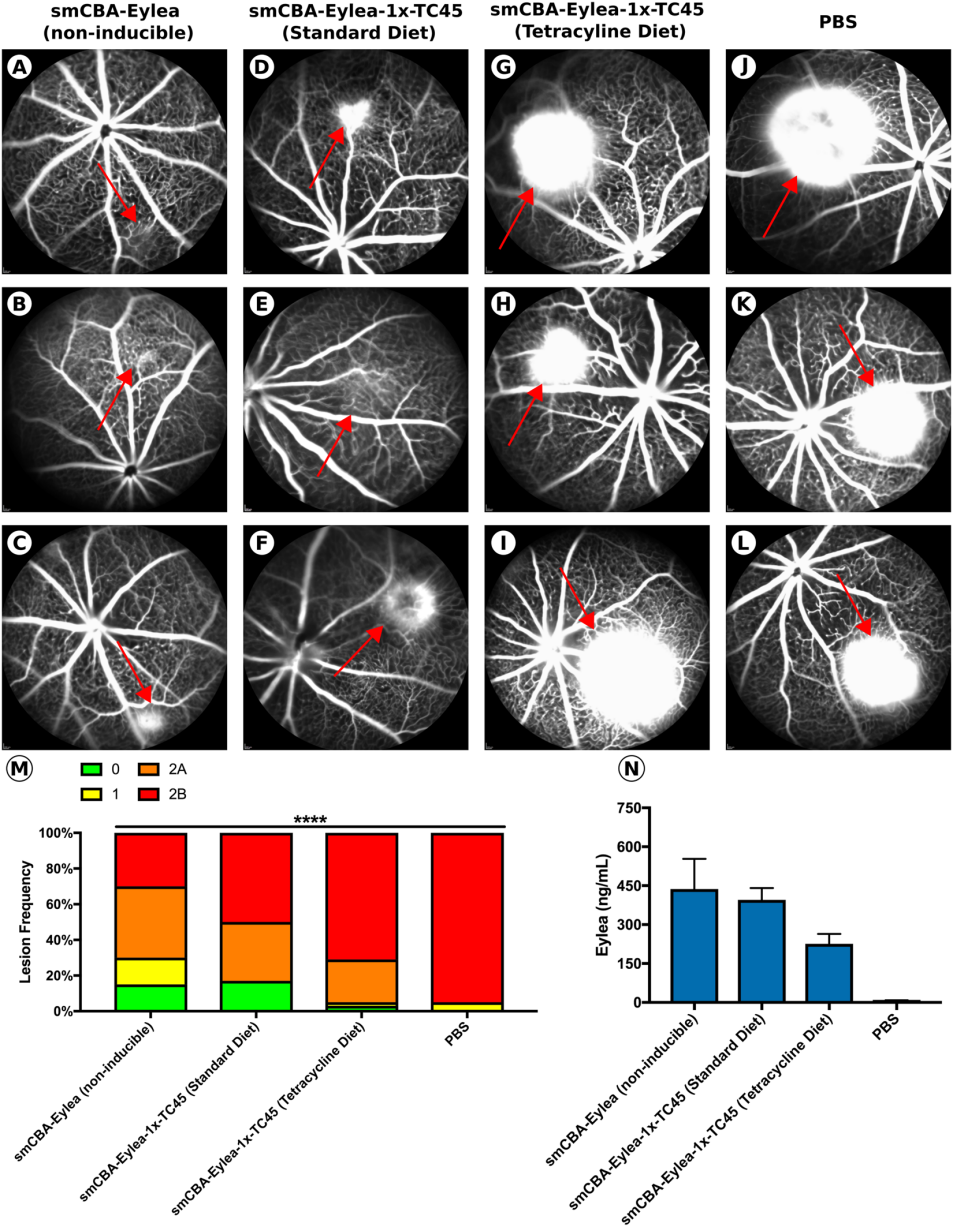
Effect of intraocular Eylea concentration on CNV lesion formation in a murine model of wet AMD. Leakage from CNV lesions was assessed 7 days following laser injury. Representative FA images taken 5 minutes after fluorescein injection for mice injected with either **(A–C)** rAAV2[MAX].smCBA-Eylea, **(D–F)** rAAV2[MAX].smCBA-Eylea-1×-TC45 + standard diet, **(G–I)** rAAV2[MAX].smCBA-Eylea-1×-TC45 + tetracycline diet or **(J–L)** PBS (*n* = 16–20 lesions per group). Images of 3 mice per group with red arrows indicating site of the laser injury. **(M)** Distribution of lesions graded independently by three masked scientists. *n* = 16–20 lesions per group, *p* < 0.0001, Chi-squared test. **(N)** Intraocular levels of non-complexed Eylea assayed by ELISA. *n* = 5 eyes per group, standard error displayed.

Next, we assessed whether delivery of a secretable Eylea transgene cassette using a rAAV capsid mutant vector with improved retinal penetrance (rAAV2/2[Y272F, Y444F, Y500F, Y730F, T491V + 7m8]; termed rAAV2/2[MAX]) is capable of preventing CNV formation following laser injury to Bruch’s membrane, a layer of extracellular matrix separating the retina from the underlying choroidal vasculature. In this model, which effectively recapitulates the exudative pathology observed in wet AMD, rupture of Bruch’s membrane results in the infiltration of new, highly permeable blood vessels into the retina in an angiogenic process mediated largely by VEGF signaling^34^. By incorporating a TC45 riboswitch in the 3′-UTR of the expression cassette, we were able modulate the intraocular concentration of Eylea through oral dosing of the activating ligand (tetracycline), allowing us to determine whether CNV lesions could be prevented in an inducible manner. To this end, age-matched C57BL/6J mice were unilaterally injected with either PBS (*n* = *10*), 1.0 × 10^10^ vg of rAAV2/2[MAX].smCBA-Eylea (Constitutive Eylea expression; *n* = *10*) or 1.0 × 10^10^ vg rAAV2/2[MAX].smCBA-Eylea-1×-TC45 (Inducible Eylea expression; *n* = *20*). Immediately following injections, half of the mice (*n* = *10*) injected with rAAV2/2[MAX].smCBA-Eylea-1×-TC45 were placed on tetracycline diet. Six weeks post-injection, CNV formation was initiated by rupturing Bruch’s membrane at two spatially discrete locations (superior and inferior retina) per eye using an infrared laser diode (810 nm, 200 mW, 75um spot size and 50 ms exposure; see methods). Seven days following laser injury, neovascular lesion size and leakage was assessed via fluorescein angiography using cSLO imaging. Lesion images from all groups were collated and randomized before being independently graded by three masked reviewers using the system described by Krzystolik *et al*., 2002 (See Supplemental Fig. 3), wherein the speed and extent of fluorescein leakage from the site of laser injury is used to determine severity, with a score of ‘2B’ being considered clinically significant (i.e. vision threatening)^35^. Ubiquitous over-expression of Eylea in rAAV2/2[MAX].smCBA-Eylea injected eyes (*n* = *10*) effectively prevented CNV formation, with an absence of lesion development at the site of laser injury (3/20 burns) or development of non-clinically significant grade 1 or 2 A lesions (3/20 and 8/20 burns, respectively) and minimal fluorescein leakage, even after a 5-minute period (Fig. 5A–C). By contrast, control eyes (*n* = *10*) injected with buffer only (PBS) predominantly developed severe grade 2B lesions (19/20 burns) characterized by rapid and extensive leakage of fluorescein into the vitreous from the site of laser injury (Fig. 5J–L). The difference in the number and distribution of lesions in rAAV2/2[MAX]smCBA-Eylea and PBS control groups was highly significant (*p* < 0.0001, Chi Square test), strongly indicating that rAAV-mediated over-expression of an anti-VEGF agent is able to prevent disease pathology in AMD (Fig. 5H).

When Eylea expression was switched off in smCBA-Eylea-1×-TC45 vector injected eyes by placing animals on tetracycline containing diet, the extent of CNV observed was comparable to PBS injected control eyes, with a substantial number of burns leading to the formation of severe grade 2B lesions (Figure G-I). By contrast, when over-expression of Eylea in rAAV2/2[MAX].smCBA-Eylea-1×-TC45 injected eyes was reduced by activation of the riboswitch, the number and severity of CNV lesions decreased dramatically, providing strong evidence that neovascularization can be prevented in an inducible manner (Fig. 5D–F). Importantly, the concentration of free Eylea protein present in each eye correlated strongly with the observed phenotype (Fig. 5N), wherein, eyes injected with non-inducible rAAV2/2[MAX].smCBA-Eylea had the highest levels of free Eylea protein (438 ng/mL). Activation (i.e. switching off) of the riboswitch in rAAV2/2[MAX].smCBA-Eylea-1×-TC45 injected eyes led to a significant (P < 0.05) 1.75-fold decrease in intra-ocular Eylea concentration (395 ng/mL to 232 ng/mL) that correlated to the substantial increase in lesion size, frequency and severity.

## Discussion

In this study we utilized a novel *in vivo* imaging approach to identify riboswitches that were capable of mediating significant changes in transgene expression when delivered to the retina using an rAAV expression vector. We subsequently demonstrated for the first time that riboswitch-mediated over-expression of an anti-VEGF agent is able to prevent CNV formation in an inducible manner following a single intravitreal injection of rAAV, potentially opening the door to a life-long, minimally invasive, but adaptable gene therapy treatment for neovascular/wet AMD.

Current treatment strategies for patients with wet AMD focus on reducing the intraocular concentration of free VEGF through repetitive monthly injections of anti-VEGF agents, a treatment paradigm that imposes a substantial medical and economic burden on the patient. Due to the invasive nature of the intravitreal injection approach and the short half-life (~7 days) of anti-VEGF agents, patients are administered an extremely large drug bolus (2 mg) per dose. This strategy results in a period of overdosing immediately post-injection, followed by a gradual decline in anti-VEGF protein levels until the concentration eventually reaches sub-therapeutic levels, at which point a further bolus is typically administered. While effective at controlling CNV formation in wet AMD, repetitive bolus dosing of anti-VEGF agents has been associated with accelerated retinal and choroidal degeneration and atrophy, leading to a progressive and significant decline in visual acuity^8,36,37^. While it remains unclear what causes acceleration of geographic atrophy, it is likely due to over-sequestration of VEGF - an essential trophic factor for both Müller glia and photoreceptor cell survival^8,37^ - following high concentration bolus administration of anti-VEGF agents. As a consequence of the invasiveness and apparent toxicity associated with repetitive intra-ocular injection of anti-VEGF agents, there is a clear need to develop a long-term, single use treatment for AMD that allows for tight temporal control of anti-VEGF expression.

Initial efforts at developing a rAAV gene therapy strategy to prevent CNV in AMD have focused on the development of platforms that reduce VEGF levels permanently through constitutive over-expression of an anti-VEGF agent. In particular, strategies aimed at limiting VEGF signaling through rAAV-mediated expression of soluble fms-like tyrosine kinase 1 (sFLT-1) have demonstrated some degree of efficacy in limiting CNV formation in pre-clinical animal models of wet AMD and have now progressed to Phase I/II clinical trials^38–41^. While the one-year follow-up of patients injected with rAAV2/2-sFLT-1 demonstrated safety of the vector, concerns still exist for potential complications of constitutive VEGF inhibition. More problematically, while 57% of patients receiving rAAV2/2-sFLT-1 maintained or improved vision, a large portion of patients still required supplemental bolus injections of anti-VEGF agents, effectively negating the benefits associated with using an rAAV-based gene therapy approach, which has been found to mediate stable levels of gene expression over a period of years^42^. It is likely the lack of therapeutic efficacy observed in many trial patients is due to the relatively low affinity of sFLT-1 for VEGF-A compared to the routinely used pharmaceutical anti-VEGF agents, such as Eylea or Lucentis^39^, resulting in sub-therapeutic sequestration of VEGF. As a consequence, we hypothesized that rAAV-mediated over-expression of Eylea – a recombinant fusion receptor that binds VEGF more efficiently than sFLT-1 – might allow for more effective long-term prevention of CNV formation without necessitating supplementation of anti-VEGF agents.

Owing to the potential for toxicity, we determined that expression of Eylea following rAAV-mediated delivery to the retina would necessarily have to be temporally regulated, allowing for periodic and controlled dosing of the anti-VEGF agent. Currently, the majority of research studies utilize inducible promoter systems to regulate transgene expression. Unfortunately, there are numerous concerns that severely limit their utility in gene therapy applications. First, such systems are typically based upon bacterially derived control elements and as such require the expression of exogenous (i.e. non-human) proteins in order to function, increasing the likelihood for immunogenicity. Second, the large size of the bacterial operon – minimally consisting of a constitutively transcribed transcriptional activator, response elements and a promoter driving expression of the therapeutic transgene – is highly problematic due to the limited coding capacity (~4.7 kb) of the rAAV vector. This factor is so problematic that it effectively prevents the inclusion of inducible gene expression systems except where the therapeutic cDNA is small (<1000 bp). While the coding sequence of Eylea is relatively small (~1,400 bp), it is still too large to package into a rAAV vector using traditional inducible promoter systems. Last, the activating ligand for these systems are currently limited to antibiotics, antimycotics and immunosuppressants, which have the potential to cause serious systemic off-target effects when provided at high enough doses to reach the retina. Although the activating ligand for the riboswitches tested in this study were also confined to suboptimal compounds (tetracycline, guanine and theophylline), the ability to design novel riboswitches that can respond to potentially any small molecule drug, protein or ion is an extremely beneficial feature of riboswitches.

Due to their small size and ability to be designed against theoretically any activating ligand, we determined that riboswitches represent a promising alternative technology for controlling gene expression levels; however, little is known about their dynamics *in vivo* and they have not previously been tested in the eye. As a consequence, we first assessed whether riboswitches are able to effectively modulate gene expression in the retina when incorporated in an rAAV expression cassette. Owing to a lack of information as to which drugs penetrate the blood-retinal barrier, we selected three ‘ON-type’ and two ‘OFF-type’ riboswitches that respond to different activating ligands (tetracycline, theophyline and guanine) that were previously isolated in work by Beilstein *et al*.^31^, Chen *et al*.^30^, Nomura *et al*.^27^, Win *et al*. (2005), and Zhong *et al*. (2017). As the number of riboswitch copies included in any expression construct has a significant effect on dynamics, we initially determined the optimal copy number for each switch using a dual-luciferase reporter system that allows precise quantification of biolumenscence levels in response to ligand supplementation. Although riboswitches have been shown to be functional when placed in either the 5′ or 3′-UTR, we chose to only evaluate these switches in the 3′-UTR in order to avoid riboswitch-independent reduction in basal expression from improper translation due to the presence of artificial start codons located in the riboswitch sequence. As ‘ON-type’ switches should be in the ‘active’ conformation in the absence of the activating ligand, and only one cleavage event is required for inhibition of translation, increasing the number of copies should also increase the likelihood of mRNA transcript cleavage occurring. As expected, increasing the number of copies for each ‘ON-type’ riboswitch (K19, L2Bulge9 and L2Bulge18tc) led to a decrease in basal expression. Moreover, the maximal expression at ligand saturating levels also decreased as copy number increased, owing to the fact that multiple copies must be inactivated in order for translation to occur. Interestingly, the same trend occurred for the two ‘OFF-type’ riboswitches (GuaM8HDV and TC45) examined, with basal expression decreasing inversely to copy number. This phenomenon is likely a result of the inherent leakiness/instability of RNA devices, wherein only a small amount of energy (<2 kcal/mol) is required to drive the transition between active and inactive confirmations, leading to improper mRNA strand cleavage even when no ligand is present^43^. Consequently, increasing the number of copies of an ‘OFF-type’ switch increases the likelihood of improper strand cleavage. As expected, gene expression decreased as copy number increased in the presence of ligand saturating levels for both ‘OFF-type’ switches. Although every riboswitch – both ‘ON-type’ and ‘OFF-type’ – exhibited a decrease in gene expression both at basal and ligand saturating levels as copy number was increased, there was no direct correlation between copy number and dynamic range, demonstrating the importance of screening switches to establish their unique dynamics.

Having determined the optimal copy number of each riboswitch, we next determined the dynamics and dose-responsiveness in cell culture using a rAAV dual-fluorescent reporter assay. Ligand dependent activation of all five riboswitches caused a significant change in GFP fluorescence in a dose dependent manner, with L2Bulge18tc and TC45 producing the largest dynamic range for ‘ON-type’ and ‘OFF-type’ switches, respectively. Notably, the dynamic range achieved in our hands for all riboswitches were consistently narrower than previously published literature, likely due to numerous experimental differences including: the cell line (varying ligand penetration), the positioning of the riboswitch within the cassette, differences in promoters and reporter transgenes (unequal protein expression and turnover), and lastly, the concentration of DNA transfected. For instance, Zhong *et al*.^29^ reported a dynamic range of 9.9-fold for 1×-TC45 in HeLa cells transfected with a cassette expressing eGFPd2, a destabilized GFP transgene (personal correspondence with Dr. Guocai Zhong) at a concentration of 0.28 pg/cell. In this study, the use of HEK293T cells (possible lower uptake of tetracycline), an unmodified GFP reporter (13x longer half-life than eGFPd2^44^), and co-transfection of an mCherry reporter to account for variations in transfection efficiency and a higher DNA concentration (10 pg/cell), likely led to the decreased dynamic range observed (4.6-fold).

In order to accurately assess changes in gene expression in the retina following rAAV-mediated delivery, we utilized a custom-built multiline cSLO instrument that is capable of discriminating the signal from GFP and mCherry fluorescent proteins^45^. Co-injection of a non-inducible vector (rAAV2/2.CBA-mCherry) allowed for normalization of images at each time point, effectively eliminating the possibility that observed changes in intra-ocular fluorescence levels were due to imaging artifacts (e.g. shadowing). Interestingly, following oral gavage of the activating ligand, all three ‘ON-type’ switches performed similarly *in vivo* and in cell culture. However, both ‘OFF-type’ switches did not reach the dynamic range achieved in cell culture, potentially owing to the relatively longer half-life of GFP, which affords for minimal GFP turnover during the 2-hour time period between oral gavage and post-treatment imaging. Continuous administration of the activating ligand (tetracycline) via dietary supplementation in rAAV2/2.smCBA.GFP-1×-TC45 injected mice increased the dynamic range from 1.5 to 2.2-fold compared to oral gavage. However, the dynamic range still did not reach the levels observed in cell culture (4.6-fold), likely due to differences in protein turnover rate, drug penetrance and clearance *in vivo* versus *in vitro*. As GFP has a long half-life (26 hours)^44^ and tetracycline clearance is relativity fast (6 hours)^46^, it is likely tetracycline concentrations in the retina never reach the required concentration to constantly saturate the TC45 riboswitch, leading to a decrease in riboswitch performance *in vivo*.

While dual-color cSLO fluorescent imaging is an effective method for monitoring fold-change in gene expression in real time, it is important to determine the absolute dynamic range of the switch by measuring protein levels, as the substantial dynamic range is essential for clinical translation. Although the protein fold-change of the best performing ‘ON and OFF-type’ were similar, the dynamic range of the TC45 switch was substantially larger than that of the L2Bulge18tc switch (227 ng/mL to 73 ng/mL, respectively). Due to its superior dynamic range, TC45 was the riboswitch chosen to modulate the expression of Eylea.

Next, we assessed whether over-expression of secretable Eylea following rAAV delivery is an effective therapeutic strategy for limiting CNV formation, using a well-established murine model of wet AMD. We utilized a rAAV2 based capsid mutant vector rAAV2/2[MAX] described by our group that is capable of efficiently transducing all layers of the neural retina following intravitreal delivery, leading to high pan-retinal levels of transgene expression^45^. Our results demonstrated that rAAV mediated over-expression of Eylea effectively limits CNV formation in wildtype mice following laser injury. Subsequently, we evaluated the efficacy of our best performing riboswitch (1×-TC45) in regulating Eylea expression following rAAV delivery to the retina. Inclusion of one copy of the TC45 riboswitch into the 3′-UTR of the Eylea transgene cassette resulted in tetracycline dependent modulation of Eylea expression and a significant change in lesion severity. Importantly, the level of free Eylea as detected by ELISA directly correlated to the frequency of clinically significant ‘Grade 2B’ lesions. While it is difficult to extrapolate dosing information from a small animal model to a human, considering that the vitreous volume of a mouse eye is approximately 1000-fold smaller than that of a human, and we detected up to 450 ng of free Eylea (i.e. unbound to VEGF) per mouse eye, it might reasonably be expected that an intraocular dose of Elyea in the low milligram (i.e. ~0.5–1.0 mg) range might be achieved in a human eye following activation of gene expression, assuming administration of an appropriately scaled rAAV vector dose. Importantly, achieving a dose in the low milligram range following riboswitch activation would place our inducible gene therapy treatment in line with current dosing guidelines, wherein patients typically receive a 2 mg bolus injection of Eylea and this is sufficient to limit CNV progression throughout a one-month period. As a consequence, we believe that inducible rAAV-mediated expression of Eylea may be a viable therapeutic strategy for the treatment of exudative AMD that eliminates the need to repetitive intravitreal injections.

Mice ubiquitously over-expressing Eylea were not observed to develop any noticeable retinal degeneration, likely owing to the short experimental time course (seven weeks) and the absence of pre-existing geographic atrophy in wild-type animals. Due to the lack of observable phenotype, we were therefore unable to address whether constitutive over-expression of Elyea causes geographic atrophy directly, or whether riboswitch-mediated downregulation of Eylea can limit the progression of retinal atrophy while also controlling CNV formation.

While TC45 activation led to a significant 1.7-fold decrease in intraocular Eylea concentration, the dynamic range was again notably lower than levels achieved in cell culture. As previously mentioned, decrease in riboswitch performance is likely a consequence of numerous factors including the long half-life of Eylea (~7 days), the high rate of tetracycline metabolism in mice, and limited uptake of tetracycline by retinal cells. In order to develop a more clinically relevant treatment, it would be beneficial to improve the dynamic range through engineering a synthetic riboswitch capable of binding a compound that is more bioavailable to retinal cells following oral dosing. Ideally, aptamers would be designed against biologically inactive compounds possessing a long half-life that have the capability of crossing the blood-retinal barrier.

Although we conducted this proof of concept experiment using an ‘OFF-type’ riboswitch due to its superior dynamic range, inclusion of an ‘ON-type’ riboswitch, where protein translation is activated upon ligand binding, would be optimal for modulating Eylea expression in a clinical setting. Specifically, insertion of an ‘ON-type’ riboswitch with low basal expression and large dynamic range would allow for periodic high-level over-expression of Eylea through oral dosing of the activating ligand, in a manner similar to the current treatment paradigm. Moreover, based on CNV progression the clinician could alter the dose or frequency of the activating ligand throughout the patient’s life in accordance with treatment needs, allowing for a personalized gene therapy approach. A secondary benefit of using an ‘ON-type’ switch is the ability to effectively turn off transgene expression by simply ceasing dosing of the activating ligand. This added safety feature could be critical for patients experiencing retinal toxicity related to long-term expression of Eylea. Unfortunately, the current library of functional ‘ON-type’ riboswitches have a narrow dynamic range in the retina, therefore limiting their use.

In order for a tunable anti-VEGF therapy for the treatment of wet AMD to be successful, it is likely that free VEGF levels will need to be tightly regulated so that they reach a steady state concentration wherein CNV formation is prevented, but retinal atrophy is not accelerated. It is possible that the cause of retinal toxicity seen in patients receiving recurring intravitreal injections is due to VEGF dropping below normal physiological levels, particularly in the days immediately following injection^37^. As retinal atrophy in patients occurs only after multiple rounds of VEGF-trap bolus injections, the cause of accelerated choroidal and retinal degeneration must be further investigated.

In summary, the work presented here demonstrated the ability to modulate Eylea expression *in vivo* following rAAV delivery through the use of the ‘OFF-type’ TC45 riboswitch. Overall, the results in this study lay the groundwork for developing a personalized gene therapy approach for the treatment of wet AMD, wherein Eylea expression can be regulated to fit the patient-specific effective dose without the need for recurring intravitreal injections.

## Methods

### Animals

Animal experiments were performed in accordance to the Association for Research in Vision and Ophthalmology (ARVO) Statement for the Use of Animals in Ophthalmic and Visual Research Guidelines and approved by the Medical College of Wisconsin Institutional Animal Care and Use Committee before beginning experiments. Four-week-old female C57BL/6J mice were obtained from Jackson Laboratory (Bar Harbor, ME) and housed in a 12-hour light and 12-hour dark environment with constant access to food and water unless otherwise stated.

### Cell culture conditions

HEK293T cells were obtained from ATCC (#CRL-11268, Manassas, VA) and cultured in Dulbecco’s Modified Eagle Medium with high glucose and GlutaMAX (Thermo Fisher Technologies, Carlsbad, CA). Media was supplemented with 1% Antibiotic-Antimycotic and 10% FBS (Gibco Life Technologies, Carlsbad, CA) unless otherwise stated. Cells were stored in a 37 °C incubator with 5% CO_2_.

### Plasmid constructs

Riboswitch constructs (K19, L2Bulge9, L2Bulge18tc, GuaM8HDV and TC45) were synthesized by GeneWiz (South Plainfield, NJ) in quadruplicate with unique 15 nucleotide barcodes flanking each copy to allow for PCR amplification of a unique number of copies; insulator sequences flanking both the 5′ and 3′-ends were also included to prevent misfolding (see Supplementary Materials and Methods). One, two, three or four copies of each construct were PCR amplified and individually cloned into the 3′-UTR of the Firefly luciferase transgene in the pmiRGLO construct (#E1330, Promega, Madison, WI) using NheI and SbfI restriction sites. The optimal copy number of each riboswitch was cloned into the 3′-untranslated region (NheI and SbfI restriction sites) of a custom-made rAAV human codon optimized GFP cassette containing a small chimeric cytomegalovirus enhancer and a chicken beta-actin (CBA) promoter driving expression. A codon optimized Eylea sequence containing the interleukin-2 secretion signal was synthesized by GeneWiz (South Plainfield, NJ). The transgene was cloned into a custom-made rAAV cassette containing a small chimeric cytomegalovirus enhancer and a chicken beta-actin promoter using NotI and SbfI restriction sites. An inducible Eylea construct was generated by replacing the GFP transgene of the smCBA-GFP-1×-TC45 cassette with Eylea using NotI and NheI restriction sites.

### Dual luciferase assay

HEK293T cells were plated in 12-well plates 24 hours prior to transfection. On the day of transfection, HEK293T cells were transfected individually with polyethylenimine (PEI) and 1 μg of each pmiRGLO construct in the presence or absence of the corresponding activating ligand in media containing 2% FBS. 24 hours after transfection, samples were harvested and processed according the manufacturer’s instructions (Dual Luciferase Reporter Systems Kit, Promega, Madison, WI). Following luminescence readings, Firefly luminescence was normalized to Renilla luminescence.

### Cell culture fluorescence assay

HEK293T cells plated in a 12-well plate were transiently transfected in transfection media with or without the corresponding activating ligand with 1μg of each GFP-riboswitch construct as well as 1μg of a non-inducible mCherry reporter. 24 hours post-transfection, representative GFP and mCherry fluorescent images were taken with a Leica inverted fluorescent microscope using a 10x objective. Following imaging, media was removed and cells were suspended in phosphate buffered saline (PBS). Fluorescent intensities were measured for GFP (485 nm excitation/528 nm emission) and mCherry (570 nm excitation/645 nm emission) using a Synergy H4 plate reader. Following data collection, GFP fluorescence was normalized to the non-inducible mCherry control.

### Recombinant adeno-associated virus production

rAAV virus was manufactured as previously described^47^. To this end, HEK293T cells were seeded in a Corning HyperFlask (New York, NY) and triple transfected using a 2:1 PEI:DNA ratio with a helper plasmid containing adenoviral elements (pHelper), a plasmid containing the *Rep* and *Cap* genes from either AAV serotype 2 (pACG2, kind gift from William Hauswirth) or a capsid mutant vector (rAAV2/2[MAX]), and finally an ITR containing plasmid to be packaged. Three days following transfection cells were harvested and lysed. Virus was purified using iodixanol ultra centrifugation and cleaned and concentrated in Hank’s Balanced Salt Solution (HBSS) + 0.014% Tween-20 using a 100 kDa centrifuge column (Amicon, Darmstadt, Germany). rAAV was titered using a picogreen assay as previously described^48^.

### Intraocular injections

Wildtype C57BL/6J mice PW4 (post-natal week 4) were sedated via an intraperitoneal injection of ketamine (60 mg/kg; Bioniche, Galway, Ireland) and xylazine (10 mg/kg; Lloyd, Shenandoah, IA). Following sedation, eyes were dilated with 2.5% phenylephrine HCl (Paragon BioTeck, Portland, OR) and 1% tropicamide (Akorn, Lake Forest, IL). For riboswitch evaluation experiments mice received a 2 μL unilateral intravitreal injection consisting of 1 μL of a rAAV2.smCBA-GFP.Riboswitch construct (1.2 × 10^12^ vg/mL) along with 1μL of rAAV2.smCBA-mCherry (1.2 × 10^12^ vg/mL) using a Microliter Syringe (Hamilton, Reno, NV) attached with a 33 G sharp needle via trans-scleral injection route. For Eylea experiments mice received a 2μL unilateral intravitreal injection of either rAAV2[MAX].smCBA-Eylea (5.0 × 10^12^ vg/mL) or rAAV2[MAX].smCBA-Eylea-1×-TC45 (5.0 × 10^12^ vg/mL) following the technique previously described.

### Confocal scanning laser ophthalmoscope imaging

A custom-made multiline confocal scanning laser ophthalmoscope (cSLO; Spectralis HRA, Heidelberg Engineering, Heidelberg, Germany) was used to image mCherry and GFP fluorescence. Mice were sedated and dilated as previously described and a glass contact lens (Cantor and Nissel, Brackley, UK) applied on the cornea. The near-infrared (NIR) reflectance mode (820 nm laser; high pass filter with transmittance above 498 nm) was used for camera alignment. mCherry fluorescence was imaged using a 561 nm laser (transmittance 582–800 nm) and GFP fluorescence was imaged using a 486 nm laser (transmittance 502–537 nm). Dioptric focus was adjusted to the depth of highest signal and single frame images were recorded with a standardized detector sensitivity of 31, 40, 50, 60, 70, 80, 90, 100 and 109 in high-resolution mode (1536 × 1536 pixels) using a 55-degree lens.

### Image normalization and analysis

mCherry expression was used to normalize pre- and post-treatment GFP expression in each eye. To this end, an ROI was applied and mean grey-scale was measured at both time points using single frame images of equal intensity. mCherry images were normalized using the ImageJ command (Process > Math > Multiply). GFP images were normalized using mCherry images as a mask (Process > Image Calculator > mCherry image × GFP image). An ROI was applied to newly generated masked GFP images, raw grey-scale was measured.

### GFP and mCherry quantification by enzyme-linked immunosorbent assays (ELISA)

Wildtype C57BL/6J mice PW4 were injected with 1 μL of a AAV2.smCBA-GFP-1×-TC45 or a AAV2.smCBA-GFP-3×-L2Bulge18tc construct (1.2 × 10^12^ vg/mL) along with 1 μL of AAV2.smCBA-mCherry (1.2 × 10^12^ vg/mL). Half the animals were placed on custom tetracycline diet and four weeks were allowed for high levels of gene expression to occur. Eyes were subsequently harvested and flash frozen using liquid nitrogen. Eyes were thawed in 100  μL of 1x Cell Extraction Buffer PTR (Abcam, Cambridge, MA) and homogenized. mCherry and GFP protein levels were quantified using commercially available mCherry-specific (ab221829) and GFP-specific (ab171581) ELISA kits (Abcam, Cambridge, MA). In each sample, GFP protein levels were normalized to mCherry protein levels to account for any injection variability.

### Laser CNV induction

Before CNV induction, mice were sedated and pupils were dilated as previously described. Mice were then placed in front of a slit-lamp and a 2.0 mm fundus contact lens (OFA 2.0 – Ocular Instruments, Bellvue, WA) placed using Systane Ultra (Alcon, Fort Worth, TX) lubricant drops. Mice were repositioned until the fundus of the injected eye was clearly visualized. An 810 nm diode laser (Iridex, Mountain View, CA) was used to induce CNV formation with the following parameters: 75 µm spot-size, 200 mW power and 50 ms exposure. A single burn was placed 2–3 disc diameters away from the optic disc with emphasis placed on avoiding large retinal vessels. CNV induction was deemed to be successful when deep retinal whitening with formation of a subretinal bubble was observed. Any lesions lacking bubble formation following laser injury were discarded from the study. 2 burn sites were placed per eye.

### Fluorescein angiography

7 days following laser injury, CNV formation was evaluated by fluorescein angiography using a 50-degree lens on a confocal scanning laser ophthalmoscope (Heidelberg Engineering, Heidelberg, Germany). To this end, mice were sedated and pupils dilated as previously described. A custom-made contact lens was applied to the surface of the cornea to improve image quality and lessen the risk for cataract formation. The mouse was placed on a custom-made imaging platform and the camera was aligned to the retinal lesions using the infrared laser (820 nm) ensuring equal illumination of the mouse fundus. Mice subsequently received a subcutaneous injection into the scruff of AK-Fluor (Akorn, Lake Forest, IL) at a concentration of 1 mg/kg. 5 minutes (±30 seconds) post-injection, high resolution fluorescent images were recorded using a 486 nm excitation laser with 502 to 537 nm band pass emission filters at a standardized detector sensitivity (60) averaged over 40 frames.

### Lesion scoring

Images of each lesion were assigned an arbitrary number and graded in masked fashion by three individuals using similar criteria specified by Krzystolik *et al*.^35^. Briefly, lesions classified as ‘Grade 0’ had no hyperfluorescence, ‘Grade I’ lesions had hyperfluorescence with negligible leakage, ‘Grade 2 A’ lesions had low levels of leakage within the lesion area and lastly, lesions classified as ‘Grade 2B’ had high levels of leakage including outside the area of the lesion.

### Unbound eylea quantification by Enzyme-linked immunosorbent assay

The concentration of intraocular unbound Eylea was measured using a commercially available Eylea-specific ELISA (Eagle Biosciences, Amherst, NH). Immediately following fluorescein angiography, animals were sacrificed via cervical dislocation. Injected eyes were removed and flash frozen using liquid nitrogen and stored in −80 °C conditions until the tissue was processed. On the day of the experiment, the tissue was thawed in 100 µL of PBS and homogenized prior to assessing Eylea concentrations following the manufacturers protocol.

### Oral gavage and custom diet

Guanine, tetracycline and theophylline (Sigma Aldrich, St. Louis, MO) were reconstituted in ddH_2_O at a concentration of 1 mg/mL. Mice were sedated via inhalation of 2% isoflurane (Akorn Pharmaceuticals, Lake Forest, IL) and received an oral gavage at a concentration of 10 mg/kg. Custom mouse diet was manufactured by Research Diets (New Brunswick, NJ) to contain 5% tetracycline hydrochloride by weight. Mice were provided with the custom diet *ad libitum* for a period of 4 weeks.

### Statistics and graphing software

A One-Way ANOVA with Tukey’s post-hoc test was used to determine the statistical significance between dosing concentrations in cell culture for each riboswitch. A one-tailed paired *t* test was applied to determine the statistical significance between fluorescence levels in baseline and post-treatment time points for injected eyes. Distribution of lesion grades was compared using a Chi-squared test, with *p* < 0.05 considered significant. All graphs and statistical analyses were produced using GraphPad Prism 7 (GraphPad Software, La Jolla, CA).

## Electronic supplementary material

Supplementary materials and figures
