# Supplementary material for: Development of an inducible anti-VEGF rAAV gene therapy strategy for the treatment of wet AMD
Source: Sci Rep. 2018 Aug 6;8:11763. doi: 10.1038/s41598-018-29726-7 (PMC6079038; doi:10.1038/s41598-018-29726-7)
Supplement: Supplementary file 1 — Supplementary materials and figures [file 41598_2018_29726_MOESM1_ESM.pdf]

SUPPLEMENTARY MATERIALS AND METHODS

Development of an inducible anti-VEGF rAAV gene therapy strategy for the treatment of wet AMD

Christopher A. Reid[1], Emily R. Nettesheim[1], Thomas B. Connor[1], Daniel M. Lipinski[‡1,2]

[1]Department of Ophthalmology, Medical College of Wisconsin, Milwaukee, WI, USA

[2] Nuffield Laboratory of Ophthalmology, University of Oxford, Oxford, UK

CORRESPONDING AUTHOR

Daniel M. Lipinski, | +1 414-955-2062 | dlipinski@mcw.edu |

Assistant Professor, Department of Ophthalmology, Medical College of Wisconsin, USA

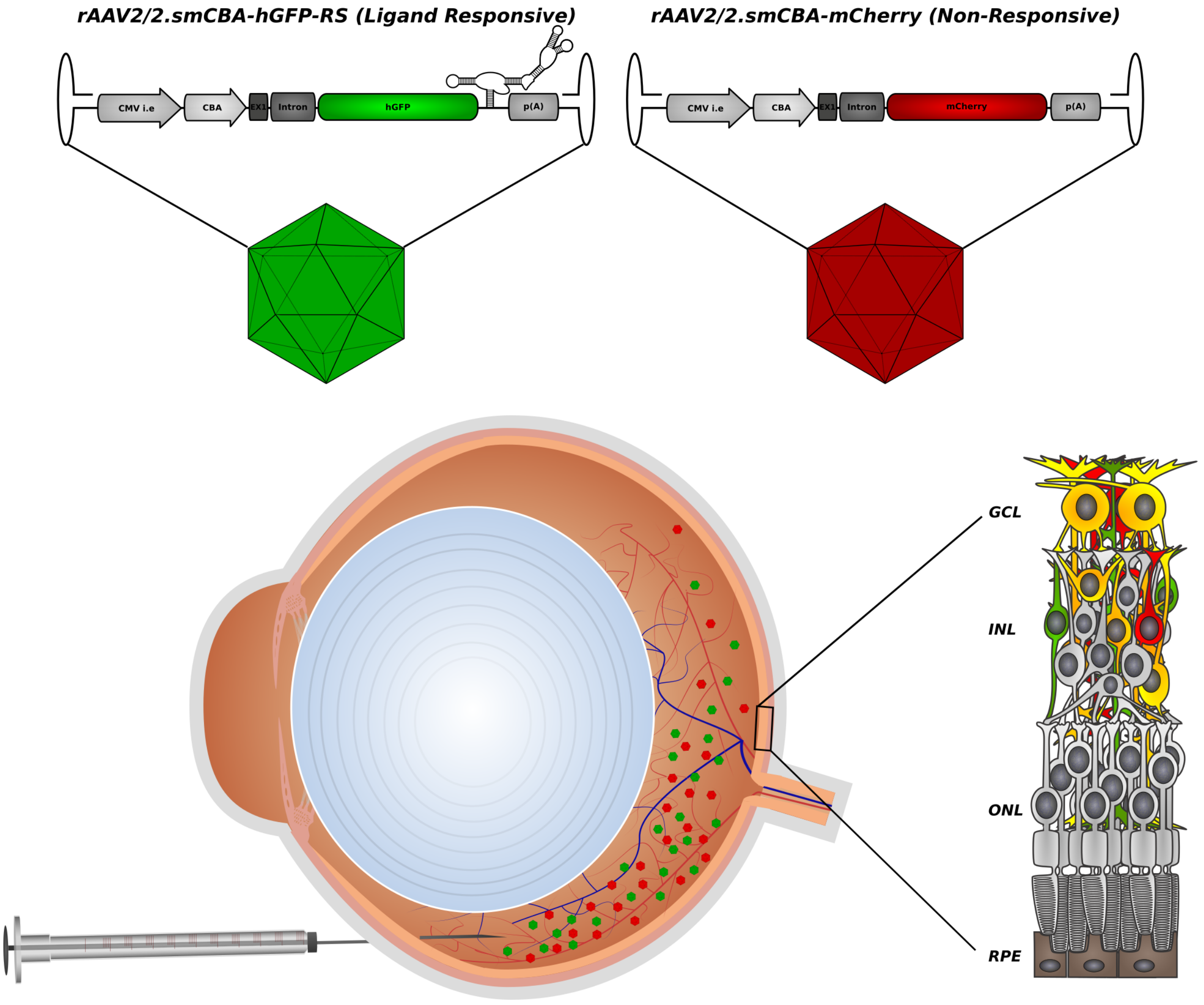

Supplemental Figure 1. Diagrammatic representative of *in vivo* experimental design. A custom made rAAV human codon optimized GFP cassette containing a small chimeric cytomegalovirus enhancer and a chicken beta-actin promoter driving expression with a riboswitch embedded in the 3'-UTR was packaged in a AAV2 capsid along with a non-inducible mCherry construct. These two rAAV vectors were intravitreally co-injected in wildtype C57BL6/J mice. Transduction from a rAAV2/2 vector delivered intravitreally is limited to the ganglion and inner nuclear cell layers.

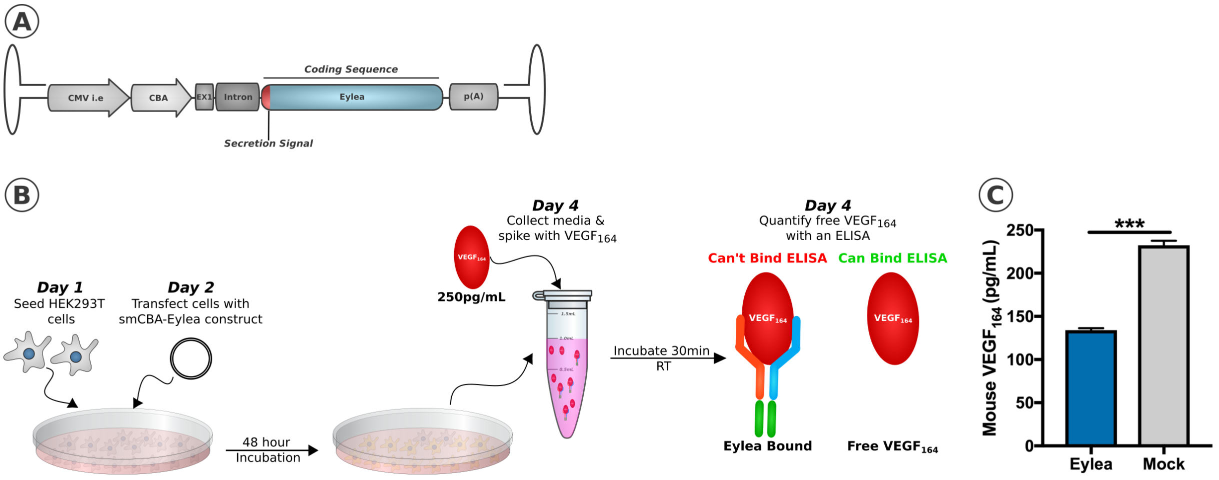

Supplemental Figure 2. Secreted Eylea successfully traps murine Vegf-A. (A) Schematic representation of the rAAV Eylea construct containing a small chimeric cytomegalovirus enhancer and a chicken beta-actin promoter driving expression of a codon optimized secreted Eylea transgene. **(B)** Diagrammatic representation of experimental conditions. **(C)** Level of unbound Vegf-A quantified by ELISA. $N = 3$ for both groups, $p = 0.0007$, unpaired t test.

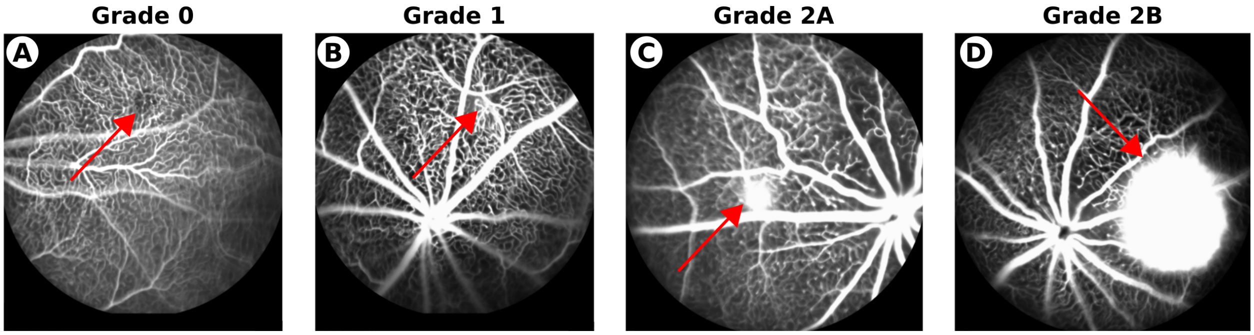

Supplemental Figure 3. CNV Lesion grading system. Representative fluorescein images taken 5 minutes after subcutaneous injection. (**A**) No hyperfluorescence or leakage at the site of laser injury (grade 0). (**B**) Hyperfluorescence at the site of laser injury with minimal fluorescein leakage (grade 1). (**C**) Hyperfluorescence and fluorescein leakage restricted to the burn area (grade 2A). (**D**) Bright hyperfluorescence and leakage beyond the site of laser injury (grade 2B).

Supplemental Table 1

**Riboswitch**	**Type**	**Aptamer**	**Ribozyme**	**Citation**
L2Bulge18tc	"ON"	Tetracycline	*sTRSV*	Chen et al.
K19	"ON"	Tetracycline	*N79 Hammerhead*	Beilstein et al.
L2Bulge9	"ON"	Theophylline	*sTRSV*	Win et al.
GuaM8HDV	"OFF"	Guanine	*Hepatitis Delta*	Nomura et al.
TC45	"OFF"	Tetracycline	*N107 Hammerhead*	Zhong et al.

Beilstein et al. (2015), Chen et al. (2010), Nomura et al. (2013), Win et al. (2005), and Zhong et al. (2017)

A - Selected riboswitch construct design

5' Restriction Site: NheI
Barcode 1: CGTATCAGCAGGTCC
Barcode 2: CCGTACGGTTCGATC
Barcode 3: GAGCTCGATGCCAAG
Barcode 4: GCATCGATCAAGCTC
Barcode 5: GATGATTCATTCGAC
3' Restriction Site: SbfI

K19-4x

ATCGGCTAGCCGTATCAGCAGGTCCCAAACAAACAAAGGCGCGTCCTGGATTCGT
GGTAAAACATACCAGATTTCGATCTGGAGAGGTGAAGAATACGACCACCTGTAGTA
TCCAGCTGATGAGTCCCAAATAGGACGAAACGCGCTAAACAAACAAACCCGTACG
GTTCGATCCAAACAAACAAAGGCGCGTCCTGGATTCGTGGTAAAACATACCAGATT
TCGATCTGGAGAGGTGAAGAATACGACCACCTGTAGTATCCAGCTGATGAGTCCC
AAATAGGACGAAACGCGCTAAACAAACAAACGAGCTCGATGCCAAGCAAACAAAC
AAAGGCGCGTCCTGGATTCGTGGTAAAACATACCAGATTTCGATCTGGAGAGGTG
AAGAATACGACCACCTGTAGTATCCAGCTGATGAGTCCCAAATAGGACGAAACGC
GCTAAACAAACAAACGCATCGATCAAGCTCCAAACAAACAAAGGCGCGTCCTGGAT
TCGTGGTAAAACATACCAGATTTCGATCTGGAGAGGTGAAGAATACGACCACCTGT
AGTATCCAGCTGATGAGTCCCAAATAGGACGAAACGCGCTAAACAAACAAAC
GATGATTCATTCGAC**CCTGCAGGATCG**

L2Bulge9-4x

ATCGGCTAGCCGTATCAGCAGGTCCAAACAAACAAAGCTGTCACCGGATGTGCTT
TCCGGTCTGATGAGTCCGTTGTCCAATACCAGCATCGTCTTGATGCCCTTGGCAGT
GGATGGGGACGGAGGACGAAACAGCAAAAAGAAAAATAAAAATTTTTTTTCCGTAC
GGTTCGATCAAACAAACAAAGCTGTCACCGGATGTGCTTTCCGGTCTGATGAGTCC
GTTGTCCAATACCAGCATCGTCTTGATGCCCTTGGCAGTGGATGGGGACGGAGGA
CGAAACAGCAAAAAGAAAAATAAAAATTTTTTTTGAGCTCGATGCCAAGAAACAAAC
AAAGCTGTCACCGGATGTGCTTTCCGGTCTGATGAGTCCGTTGTCCAATACCAGCA
TCGTCTTGATGCCCTTGGCAGTGGATGGGGACGGAGGACGAAACAGCAAAAAGAA
AAATAAAAATTTTTTTTGCATCGATCAAGCTCAAACAAACAAAGCTGTCACCGGATG
TGCTTTCCGGTCTGATGAGTCCGTTGTCCAATACCAGCATCGTCTTGATGCCCTTG
GCAGTGGATGGGGACGGAGGACGAAACAGCAAAAAGAAAAATAAAAATTTTTTTT
GATGATTCATTCGAC **CCTGCAGGATCG**

L2Bulge18tc-4x

ATCGGCTAGCCGTATCAGCAGGTCCAAACAAACAAAGCTGTCACCGGATGTGCTT
TCCGGTCTGATGAGTCCGTTGTCCAAAACATACCAGATTTCGATCTGGAGAGGTGA
AGAATTCGACCACCTGGACGAGGACGGAGGACGAAACAGCAAAAAGAAAAATAAA
AACCGTACGGTTCGATCAAACAAACAAAGCTGTCACCGGATGTGCTTTCCGGTCTG
ATGAGTCCGTTGTCCAAAACATACCAGATTTCGATCTGGAGAGGTGAAGAATTCGA
CCACCTGGACGAGGACGGAGGACGAAACAGCAAAAAGAAAAATAAAAAGAGCTCG

ATGCCAAGAAACAAACAAAGCTGTCACCGGATGTGCTTTCCGGTCTGATGAGTCC
GTTGTCCAAAACATACCAGATTTCGATCTGGAGAGGTGAAGAATTCGACCACCTGG
ACGAGGACGGAGGACGAAACAGCAAAAAGAAAAATAAAAAGCATCGATCAAGCTC
AAACAAACAAAGCTGTCACCGGATGTGCTTTCCGGTCTGATGAGTCCGTTGTCCAA
AACATACCAGATTTCGATCTGGAGAGGTGAAGAATTCGACCACCTGGACGAGGAC
GGAGGACGAAACAGCAAAAAGAAAAATAAAAAGATGATTCATTCGAC
CCTGCAGGATCG

GuaM8HDV-4x:

ATCGGCTAGCCGTATCAGCAGGTCCATGGCCGGCATGGTCCCAGCCTCCTCGCT
GGCGCCGGCTGGGCAATGCTATAATCGCGTGGATATGGCACGCAAGTTTCTACCG
GGCACCGTAAATGTCCGACTAGTAGCGAATGGGACGCACAAATCTCTCTAGCCGT
ACGGTTCGATCATGGCCGGCATGGTCCCAGCCTCCTCGCTGGCGCCGGCTGGGC
AATGCTATAATCGCGTGGATATGGCACGCAAGTTTCTACCGGGCACCGTAAATGTC
CGACTAGTAGCGAATGGGACGCACAAATCTCTCTAGGAGCTCGATGCCAAGATGG
CCGGCATGGTCCCAGCCTCCTCGCTGGCGCCGGCTGGGCAATGCTATAATCGCG
TGGATATGGCACGCAAGTTTCTACCGGGCACCGTAAATGTCCGACTAGTAGCGAA
TGGGACGCACAAATCTCTCTAGGCATCGATCAAGCTCATGGCCGGCATGGTCCCA
GCCTCCTCGCTGGCGCCGGCTGGGCAATGCTATAATCGCGTGGATATGGCACGCA
AGTTTCTACCGGGCACCGTAAATGTCCGACTAGTAGCGAATGGGACGCACAAATC
TCTCTAG GATGATTCATTCGAC**CCTGCAGGATCG**

TC45-4x

ATCGGCTAGCCGTATCAGCAGGTCCAAACAAACAAACTGAGATGCAGGTACATCC
CACTGATGAGTCCCAAATAGGACGAAAGGGAGAGGTGAAGAATACGACCACCTAG
GCTCGAAAGAGCCTAAAACATACCTTCTGGGATTCCACTGCTATCCACAAAAAGAA
AAATAAAAACCGTACGGTTCGATCAAACAAACAAACTGAGATGCAGGTACATCCCA
CTGATGAGTCCCAAATAGGACGAAAGGGAGAGGTGAAGAATACGACCACCTAGGC
TCGAAAGAGCCTAAAACATACCTTCTGGGATTCCACTGCTATCCACAAAAAGAAAA
ATAAAAAGAGCTCGATGCCAAGAAACAAACAAACTGAGATGCAGGTACATCCCACT
GATGAGTCCCAAATAGGACGAAAGGGAGAGGTGAAGAATACGACCACCTAGGCTC
GAAAGAGCCTAAAACATACCTTCTGGGATTCCACTGCTATCCACAAAAAGAAAAAT
AAAAAGCATCGATCAAGCTCAAACAAACAAACTGAGATGCAGGTACATCCCACTGA
TGAGTCCCAAATAGGACGAAAGGGAGAGGTGAAGAATACGACCACCTAGGCTCGA
AAGAGCCTAAAACATACCTTCTGGGATTCCACTGCTATCCACAAAAAGAAAAATAAA
AA GATGATTCATTCGAC**CCTGCAGGATCG**
